# DNA methylation and Transcriptome Changes Associated with Cisplatin Resistance in Ovarian Cancer

**DOI:** 10.1038/s41598-017-01624-4

**Published:** 2017-05-04

**Authors:** Riikka J. Lund, Kaisa Huhtinen, Jussi Salmi, Juha Rantala, Elizabeth V. Nguyen, Robert Moulder, David R. Goodlett, Riitta Lahesmaa, Olli Carpén

**Affiliations:** 1 Turku Centre for Biotechnology, University of Turku and Åbo Akademi University, Turku, Finland; 2 Department of Pathology, Medicity Research Unit, University of Turku and Turku University Hospital, Turku, Finland; 3 Department of Pharmaceutical Sciences, University of Maryland, Baltimore, MD USA

## Abstract

High-grade serous ovarian cancer is the most common ovarian cancer type. Although the combination of surgery and platinum-taxane chemotherapy provide an effective treatment, drug resistance frequently occurs leading to poor outcome. In order to clarify the molecular mechanisms of drug resistance, the DNA methylation and transcriptomic changes, associated with the development of drug resistance in high-grade serous ovarian cancer, were examined from patient derived malignant ascites cells. In parallel with large-scale transcriptome changes, cisplatin resistance was associated with loss of hypermethylation at several CpG sites primarily localized in the intergenic regions of the genome. The transcriptome and CpG methylome changes in response to cisplatin treatment of both sensitive and resistant cells were minimal, indicating the importance of post-translational mechanisms in regulating death or survival of the cells. The response of resistant cells to high concentrations of cisplatin revealed transcriptomic changes in potential key drivers of drug resistance, such as *KLF4*. Among the strongest changes was also induction of *IL6* in resistant cells and the expression was further increased in response to cisplatin. Also, several other components of IL6 signaling were affected, further supporting previous observations on its importance in malignant transformation and development of drug resistance in ovarian cancer.

## Introduction

High-grade serous ovarian cancer (HGSOC) is the most common ovarian cancer subtype and accounts for 80% of the deaths caused by the disease. The prognosis of HGSOC is poor as most diagnosis is at late stages of the disease when the 10-year survival rate is only in the order of 15%. The main strategy for treatment involves surgical removal of the tumor tissue and chemotherapy^1^. Platinium compounds, such as cisplatin, in combination with taxane are typically used in chemotherapy. However, recurrence of the cancer is frequent and most of the patients will eventually become refractory to the treatment^2^. In order to improve the prognosis of the patients with HGSOC, new biomarkers enabling early diagnosis of the disease as well as new therapeutic strategies overcoming the drug resistance are needed^1, 3^. Detailed characterization of the molecular mechanisms leading to drug resistance is important for development of improved therapies.

The molecular mechanisms leading to drug resistance can be heterogeneous and complex^4^. In addition to genetic factors, the development may involve epigenetic changes, which enable tumor cells, and possibly non-transformed cells in the microenvironment, to adapt and lose sensitivity to drug treatment. DNA methylation and transcriptional changes associated with drug resistance have been detected in several genomic sites in both cell lines and patient samples^5–8^. For example, methylation and transcriptional silencing of the MLH1 gene have been repeatedly associated with cisplatin resistance^8, 9^. Although several candidate driver genes for cisplatin resistance have been identified, further studies are required to clarify the heterogeneity of the drug resistance mechanisms and clinical significance of the findings.

In this study, we have further investigated the potential mechanisms associated with drug resistance by comparing cisplatin responses in sensitive and resistant patient derived HGSOC cell lines with next-generation sequencing based applications. We have used Reduced Representation Bisulfite Sequencing (RRBS) together with messenger RNA sequencing (mRNA-seq) for unbiased identification of the DNA methylation changes at single nucleotide resolution in the CpG rich regions of the genome in correlation with genome-wide transcriptome changes.

## Results

### Differences between cisplatin sensitive and resistant cells before drug treatment

**Figure 1:**
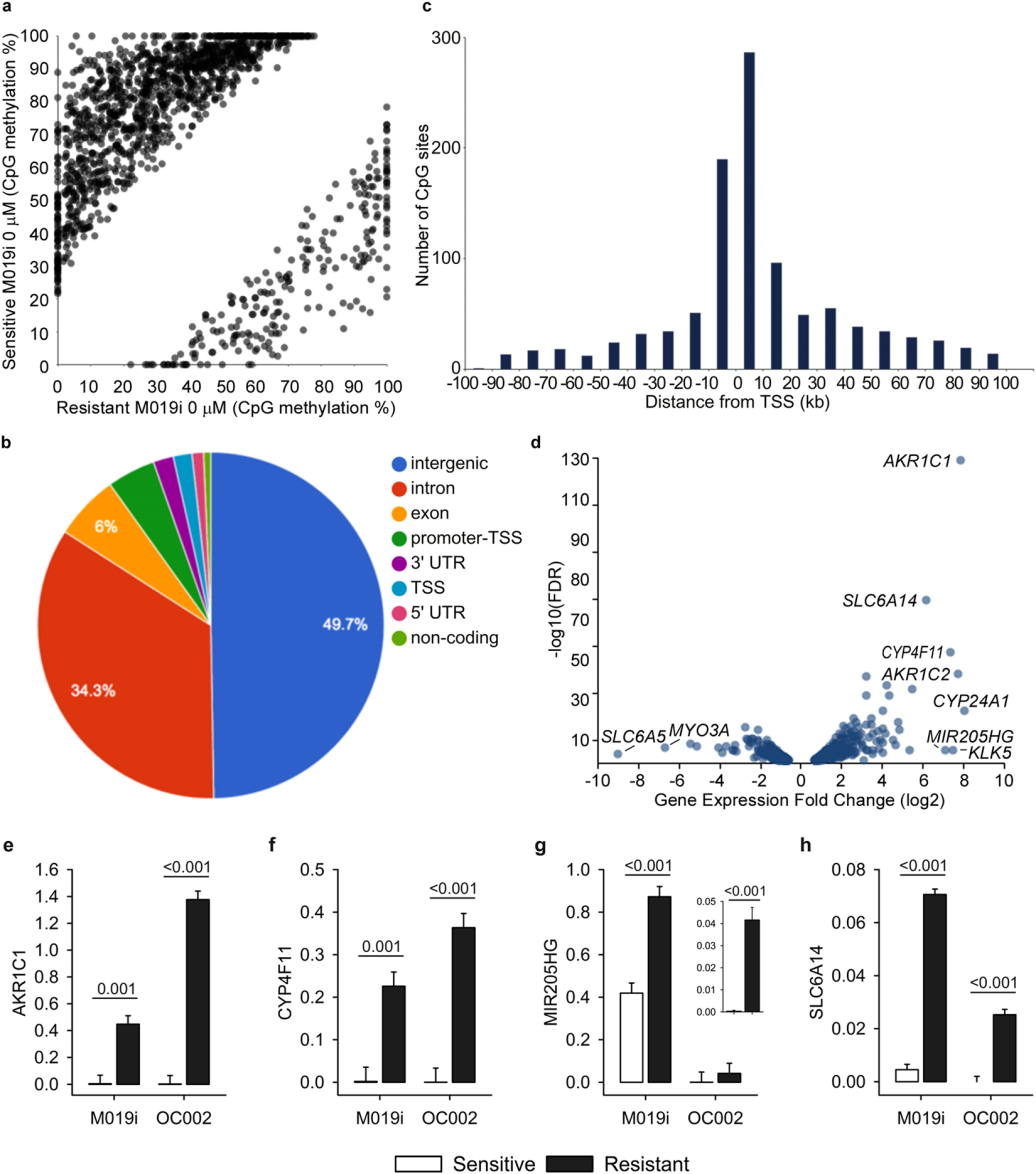
CpG methylome and transcriptome differences between cisplatin sensitive and resistant ovarian cancer lines. DNA methylomes of the cells sensitive or resistant to cisplatin were profiled with Reduced Representation Bisulfite Sequencing and transcriptomes with mRNA-sequencing. In (**a**) are the CpG sites with coverage ≥ 10 and minimum methylation difference of 20% (qval ≤ 0.05) in M019i cells, (see also Supplementary Table S1), (**b**) the distribution of differentially methylated cites in genomic regions, (**c**) distance of the differentially methylated sited from the closest transcription start sites, (**d**) the transcriptome differences (minimum absolute FC = 1.5, FDR ≤ 0.05) between cisplatin sensitive and resistant ovarian cancer cells (M019i), (**e–h**) qRT-PCR validation of *AKR1C1*, *CYP4F11*, *CYP24A1*, *MIR205HG*, and *SLC6A14* differences in M019i and OC001 cells (y-axis: relative expression level).

Comparison of cisplatin sensitive and resistant M019i cells before the drug treatment revealed large scale differences in both transcriptomes and DNA methylomes. Comparison of DNA methylomes revealed a total of 1,488 differentially methylated sites that exceeded a minimum methylation difference of 20% in each comparison (Fig. 1a, Supplementary Table S1). Interestingly, most of the differentially methylated sites (1,251 sites, 84%) were found to be less methylated in the resistant cell line. Only 237 (16%) sites were methylated at higher levels in the resistant cell line and had lower methylation levels in the sensitive line. Most of the differentially methylated sites were in the non-coding regions of the genome (Fig. 1b). Of the sites 90 (6.0%) were in exons and 26 (1.7%) in the TSS. The majority of differentially methylated sites (76%) were located within 100 Kbp distance from a TSS (Fig. 1c) and nearly all sites (1,479) were within 1 Mbp distance from a TSS of a gene. The genes close to the differentially methylated sites were associated with canonical pathways such as cAMP-mediated signaling (32 molecules, p = 7.14E-04), G-protein coupled receptor (GPCR) signaling (37 molecules, p = 8.33E-04), WNT/beta-catenin signaling (25 molecules, p = 1.92E-03) and human embryonic stem cell pluripotency (22 molecules, p = 2.11E-03), see Supplementary Table S2 for the complete list of functional enrichment results. Consistently, the top putative upstream regulators included such as POU5F1 (57 targets, p = 2.21E-10), CTNNB1 (95 targets, p = 1.42E-09), SOX2 (54 targets, 3.05E-09), KLF4 (41 targets, 6.03E-07) and TP53 (151 targets, p = 6.42E-07). However, no transcription counts were detected for well-known pluripotency factors POU5F1 and SOX2. The top molecular functions of the genes included differentiation of cells (422 molecules, 4.08E-27), proliferation of cells (604 molecules, p = 4.45E-23) and the strongest disease association was cancer (1,585 molecules, p = 2.98E-51).

Considerable differences in the transcriptome were also observed between cisplatin sensitive and resistant cells. Altogether 587 differentially expressed genes were detected. Out of these 346 showed increased expression and 241 decreased expression in the resistant cells (Fig. 1d, Supplementary Table S3). As patient characteristics may influence the results^10^, the expression changes of selected genes were further validated using qRT-PCR in two platinum sensitive cell lines M019i and OC002 and their resistant variants (Fig. 1e). Pathway analysis revealed functional enrichment of the molecules to several canonical pathways, the most prominent ones were involved in oxidative metabolism and stress, such as “production of nitric oxide and reactive oxygen species in macrophages” (19 molecules, p = 9.77E-07), and inflammatory signaling, such interferon signaling (8 molecules, p = 3.61E-06). See Supplementary Table S2 for the complete list of enrichment results. Among the most significant putative upstream regulators were TNF (132 targets, 1.19E-34), beta-estradiol (118 targets, 1.19E-34) and IFNL1 (26 targets, 4.14E-24). The genes differentially expressed by the cisplatin sensitive and resistant cells were enriched in the top functional categories including cell death (222 molecules, p = 1.72E-18) and cellular movement (160 molecules, p = 4.59E-18). Cancer was again among the most significant disease enrichments (516 molecules, p = 9.74E-15).

### Responses of sensitive and resistant cells to low cisplatin treatment

We next examined the epigenetic and transcriptional perturbations in sensitive and resistant cell lines in response to 0.6 μM cisplatin treatment. Interestingly, although cisplatin treatment led to CpG methylation changes in both sensitive and resistant cells, these alterations did not trigger changes in the gene expression. In the sensitive line, changes in the methylation of 59 sites (FDR 5%), 28 with increased and 31 with decreased methylation, were detected in response to cisplatin treatment (Supplementary Table S1). The genes closest to the altered sites were functionally linked to neuronal signaling pathways, such as development of neurons (18 molecules, p = 6.27E-06) and canonical pathways, such as synaptic long term depression (6 molecules, p = 4.11E-02). Consistently, the putative upstream regulators included APP (13 targets, p = 1.15E-04) and APOE (5 targets, p = 3.54E-03). However, no changes in gene expression were observed with the chosen filtering criteria (minimum absolute FC = 1.5, FDR ≤ 0.05 or unadjusted pval ≤ 0.05) indicating that the detected DNA methylation changes did not cause changes in transcriptional activity in sensitive cells.

In the resistant cell line changes were observed in 74 sites (FDR 5%): 34 with increased and 40 decreased methylation in response to 0.6 μM cisplatin treatment. The genes closest to the altered sites were linked to the WNT/beta-catenin canonical pathway (5 molecules, p = 2.96E-02) and, similarly to sensitive cells, in molecular functions associated with neuronal development and functions, such as development of neurons (21 molecules, p = 4.9E-06). The putative upstream regulators included REST (5 targets, p = 6.38E-04), FGFR2 (5 targets, p = 7.39E-04), CTNNB1 (11 targets, p = 1.77E-03) and KLF4 (6 targets, p = 2.72E-03). Interestingly, none of the sites differentially methylated in response to cisplatin in resistant cells overlapped with those detected in sensitive cell line. Also, as was the case for the sensitive cells, no changes in gene expression were observed in response to 0.6 μM cisplatin treatment of resistant cells (minimum absolute FC = 1.5, FDR ≤ 0.05 or unadjusted pval ≤ 0.05).

### Response of resistant cells to high cisplatin treatment

We also examined the response of the resistant cell line to a higher concentration of cisplatin (7 μM, IC50), which was not tolerated by the sensitive cells. In contrast to the lower concentration, changes were now detected in both CpG methylomes and transcriptomes. Decreased methylation of 28 sites was observed, whereas 49 sites become more methylated (Supplementary Table S1). The genes closest to the altered sites were associated with molecular functions, such as development of embryo, tissues and cells. The most prominent disease function was cancer (p ≤ 0.05), and notably, among the most significant subclasses was epithelial cancer (106 molecules, p = 3.38E-06). The canonical pathway enrichments included WNT/beta-catenin signaling (6 molecules, p = 3.54E-04).

With the harsher cisplatin treatment (7 μM) changes in transcription of 387 genes were detected (327 upregulated and 60 downregulated genes). The strongest functional and disease enrichment categories for the altered genes included cell cycle progression (61 molecules, p = 1.66E-10) and cancer (351 molecules, p ≤ 2.91E-03). The top canonical pathway enrichments indicated changes in DNA damage and cell cycle control and included such as “Role of BRCA1 in DNA damage Response” (10 molecules, p = 9.81E-07) and “Role of CHK Proteins in Cell Cycle Checkpoint Control” (7 molecules, p = 4.48E-05). The putative upstream regulators included such as let-7 (21 targets, p = 1.76E-14), TP53 (61 targets, p = 8.76E-13) and many other factors (Supplementary Table S2).

### Identification of putative drug resistance genes through integrative analysis

**Figure 2:**
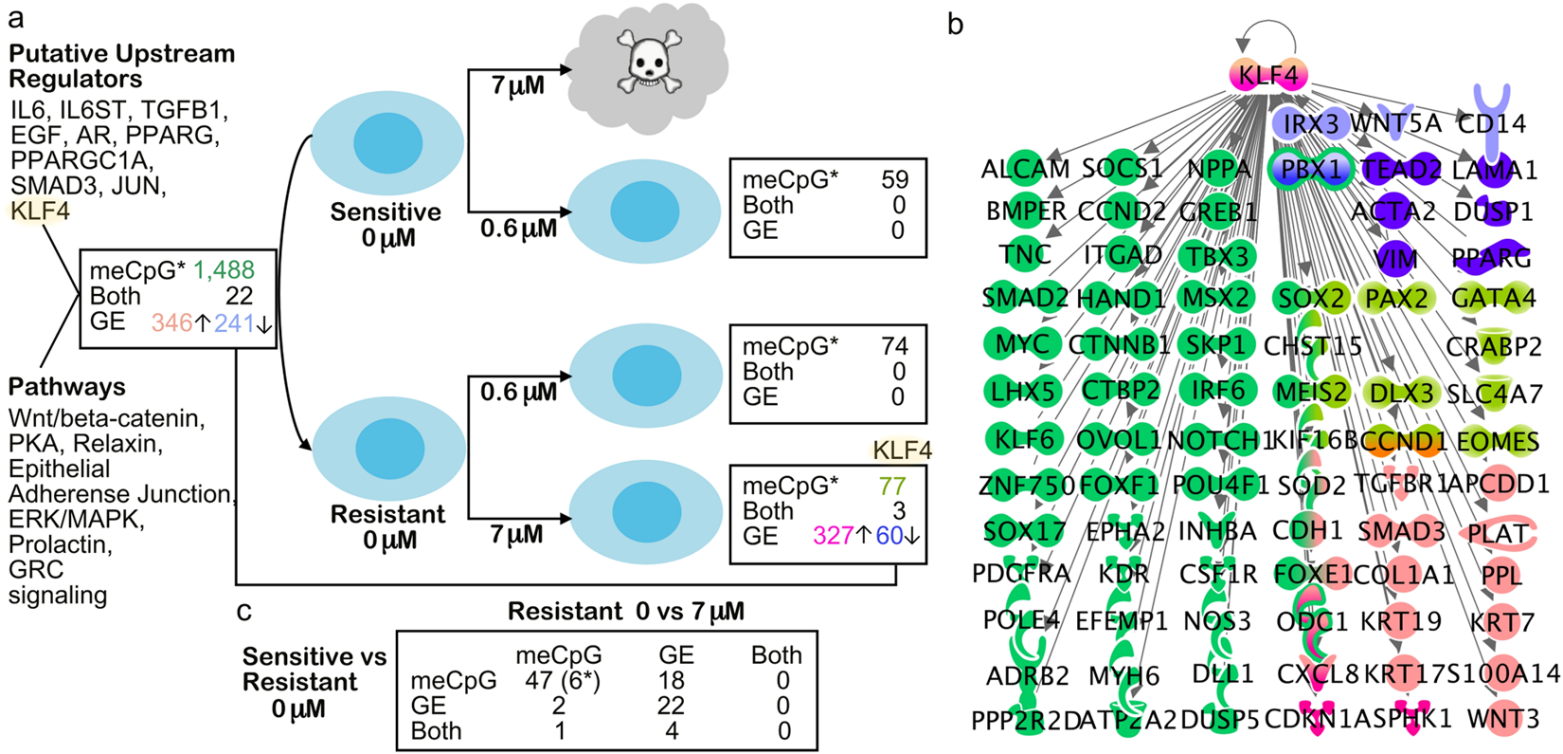
Integrative analysis of DNA methylome, transcriptome and functional enrichment data. (**a**) In the figure are the numbers of differentially expressed genes (GE), differentially methylated CpG sites (meCpG) and their overlap (both) between cisplatin sensitive and resistant cells (M019i) before cisplatin treatment (0 μM), and changes detected in response to drug treatment. The putative upstream regulators with gene expression changes and canonical pathway enrichments common for both DNA methylome and transcriptome data are shown in the (**b**) Gene expression changes, DNA methylation changes or both are shown for the known direct target genes of KLF4, in different comparisons as indicated by the color codes in (**a**). (**c**) Comparison of differences observed before cisplatin treatment and in response to 7 μM cisplatin treatment of resistant cells as indicated in the figure. *indicates the number of differentially methylated sites: in integrative comparisons, the number of overlapping genes closest to differentially methylated sites is shown. The functional analyses and networks in the figure were generated by using Ingenuity Pathway Analysis (IPA®, Qiagen).

In order to obtain deeper insights in to the molecular mechanisms of drug resistance we carried out an integrative analysis of the RRBS and RNA-seq data as well as functional enrichment data throughout the conditions (Fig. 2a). Comparison of the data sets from the cisplatin sensitive and resistant cells before drug treatment revealed overlap in 50 differentially expressed genes with a total of 90 differentially methylated CpG sites in the close genomic proximity (Supplementary Table S4). Of these CpG sites 71 (79%) were localized in functional genomic elements including exons, introns or regulatory elements, such as enhancers (annotated in ovary by Roadmap Epigenomics Project, http://egg2.wustl.edu/). Pathway analysis revealed that 22 of these differentially expressed genes associated with DNA methylation changes were involved in cell death (p = 1.00E-03) and 46 were associated with abdominal cancer (p = 7.19E-04). Comparison of the functional enrichment results, determined separately for DNA methylome and transcriptome data, revealed overlaps in the canonical pathway enrichments including WNT/beta-catenin, protein kinase A (PKA), relaxin, epithelial adherence junction, ERK/MAPK, prolactin and glucocorticoid receptor (GRC) signaling. Putative upstream regulators common for both transcriptome and DNA methylation changes, and showing changes also in gene expression between cisplatin sensitive and resistant cells, included IL6, IL6ST, SMAD3, KLF4, TGFBR1, EGF, JUN, PPARG, PPARGC1A and AR (p < 0.05) (Fig. 2a).

Integrative analysis of the DNA methylation and transcriptome changes in response to 7 μM cisplatin treatment of the resistant cells revealed only three common genes, *DUSP10, NGFR*, *NXPH3*. No overlaps in canonical pathway enrichments were observed for the transcriptome and DNA methylome data. The only common putative upstream regulator of the DNA methylation and gene expression changes was KLF4 (Fig. 2a). Interestingly, this gene was expressed more by the cisplatin resistant cells in comparison to sensitive cells already before drug treatment (5.21-fold, p = 1.59E-06) and was further induced in the resistant line in response to 7 μM cisplatin (2.28-fold, p = 3.61E-09) which was also validated using qRT-PCR (Supplemetary Figure S1). Importantly, KLF4 was also identified as a common putative upstream regulator of the genes a) differentially methylated by untreated cisplatin sensitive and resistant cells (41 targets, p = 6.03E-07) b) differentially expressed by cisplatin sensitive and resistant cells (9 targets, 1.84E-02), c) differentially methylated in response to 0.6 μM cisplatin (6 targets, p = 2.72E-03), d) differentially methylated in response to 7 μM cisplatin treatment of resistant cells (10 targets, p = 4.77E-07) and e) differentially expressed by cisplatin treated and non-treated cells (9 targets, p = 1.84E-02) (Fig. 2b). The putative target genes of KLF4 were associated with functions, such as invasion of cells (42 of 79 molecules, p = 3.73E-33), migration (49 of 79 molecules, p = 1.23E-25) and development (60 of 79 molecules, p ≤ 1.55E-11).

**Figure 3:**
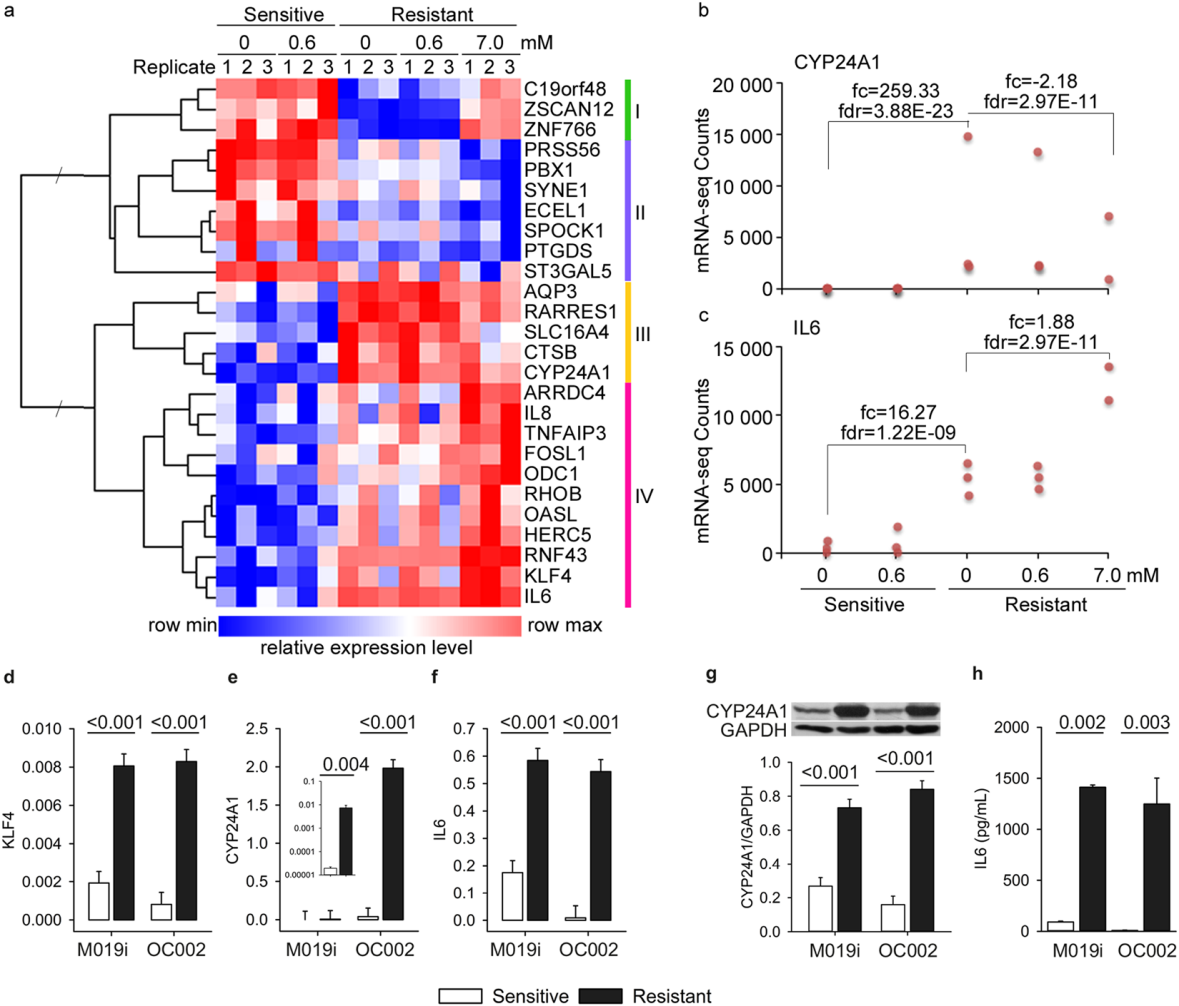
Identification of genes potentially associated with the drug resistance in ovarian cancer cells through integrative analysis. (**a**) Normalized relative gene expression counts and clustering of the genes differentially expressed by the cisplatin sensitive and resistant cells and regulated in response to 7 μM cisplatin treatment in resistant cells (M019i). (**b**,**c**) Normalized gene expression counts (RPKM) and statistics for *CYP24A1* and *IL6* genes in M019i cells. (**d**–**f**) Expression of *KLF4*, *CYP24A1* and *IL6* was validated with qRT-PCR in two platinum sensitive cell lines M019i and OC002 and their resistant variants (y-axis: relative expression level). (**g**) CYP24A1 protein expression and (**h**) IL6 protein concentrations in cell culture supernatants of the same cell lines as in (**e** and **f**), respectively).

Next we examined the regulation patterns of genes that were differentially expressed in sensitive and resistant lines and that also responded to the 7 μM cisplatin treatment in the resistant cell line. Altogether 26 such genes were identified (Fig. 2c). Of these, eight were differentially expressed by sensitive and resistant cell lines, however, the levels reverted to or towards the levels in sensitive cells in response to 7 μM cisplatin treatment (Fig. 3a, clusters I and III). Notably, among these was *CYP24A1* with barely detectable levels of expression in sensitive cells and high expression levels in resistant cells (average difference 259.33-fold). The 7 μM cisplatin treatment decreased the levels of *CYP24A1*, however, only on average by 2.18-fold (Fig. 3b) and therefore not sufficient to reach the levels observed in the sensitive cells. We further validated CYP24A1 expression at both the mRNA (Fig. 3d) and protein level (Fig. 3e). Most interestingly a panel of 18 genes was differentially expressed by normal and resistant lines and this difference was further increased by the drug treatment of resistant line (Fig. 3a, clusters II and IV). The gene with the strongest difference was *IL6* (Fig. 3c) with expression pattern similar to *KLF4* (correlation 0.96), a known upstream regulator of IL6. Consistently with the mRNA data, the IL6 protein levels produced by the platinum resistant cells were significantly higher than by the sensitive cells (Fig. 3f). Further, several other components of the IL6 signaling or network were differentially expressed by the sensitive and resistant lines and affected by 7 μM cisplatin treatment (Supplementary Table S2). In cluster II, among the genes with opposite pattern of expression, *PBX1* had the strongest negative correlation (−0.86) with *IL6*. The regulation of *IL6* and *CYP24A1* in resistant cells was reciprocal. Interestingly, functional enrichment analysis revealed strong association of these genes with abdominal cancer (24 of 26 molecules, p = 1.78E-5). Furthermore, most of these genes have previously been associated with ovarian cancer, and several with malignancy or drug resistance (Table 1), supporting the potential importance of these genes as key drivers of drug resistance in ovarian cancer^11–30^.

**Table 1:** The potential drug resistance driver genes.

Gene	RNA-seq (FC, FDR ≤ 0.05)		Closest DMS and distance (Kbp)	RRBS (meDiff%, qval ≤ 0.05)	Previous Links to Ovarian Cancer
	Sens 0 μM vs Res 0 μM	Res 0 μM vs Res 7 μM		Sens 0 μM vs Res 0 μM	
AQP3	5.9	−1.86	chr9:36166020–2,725	−70	Regulates cell migration in EGF dependent manner^18^
CTSB	5.09	−1.84	chr8:10897405–803	−50	Unfavorable marker for survival^19^
CYP24A1	259.33	−2.18	chr20:52556813–213	−67	Increased expression^20, 21^
PRSS56	−2.94	−2.38	chr2:232290776–1,094	−57	—
ECEL1	−3.46	−2.06	chr2:232290776 1,054	−57	Chemotherapy-resistance^22^
SPOCK1	−3.29	−2.65	chr5:133477187 2,834	−54	Growth and metastasis^23^
SYNE1	−2.17	−2.22	chr6:156200288–3,242	−71	Mutations predictive for malignant transformation^24, 25^
PBX1	−2.09	−1.97	chr1:164500920–28	−46	Mediated survival in response to Notch3^26^
PTGDS	−3.79	−2.08	chr9:136310987 168	68	Chemoresistance^33^
ST3GAL5	−2.41	−2.02	chr2:85892782 173	−37.61	Decreased expression^27^
FOSL1	1.98	1.98	chr11:65682138–14	−43	ER-dependent induction impaired in response to saracatinib+/− fulvestrant^28^
IL8/CXCL8	1.74	2.14	chr4:68449172–6,157	−44	Chemoresistance^33^
ARRDC4	1.95	2.1	chr15:98937633 421	47	—
TNFAIP3	3.33	2.46	chr:136929632–1,259	−63	Induced by ARID3B^29^
ODC1	2.05	1.94	chr2:10445016 136	−65	Downregulated in response to platinium drugs^30^
RNF43	3.63	1.66	chr17:55185542 1,245	−34	Tumor supressor and mutation hotspot^51, 52^
HERC5	2.41	1.74	chr4:68449172–20,929	−44	—
OASL	4.99	1.93	chr12:121177842 280	−56	Chemoresistance^33^
KLF4	5.21	2.28	chr9:115618930–5,367	−48	Downregulated and regulates BCL2/Bax ratio^53^, mediates EMT^54^
IL6	16.27	1.8	chr7:30737735–7,971	−47	Chemoresistance^33^, potential prognostic marker^32, 34^

Correlation to the CpG methylation changes revealed that nine genes including *CTSB*, *CYP24A1*, *PBX1*, *PTGDS*, *ST3GAL5*, *FOSL1, ARRDC4*, *ODC1* and *OASL* had differentially methylated sites within 1,000 Kbp distance from the gene when comparing the untreated resistant to the sensitive cells (Table 1). Treatment with cisplatin did not induce consistent DNA methylation changes in these sites, except at the site close to the *CTSB* gene.

In summary, the drug resistance of the ovarian cancer cells was associated with large-scale changes in the regulation of *KLF4* target genes and with gene expression changes in a subset of genes, including *IL6*, for which the differences between sensitive and resistant cells were further potentiated in response to 7 μM cisplatin treatment.

### Comparison to previous studies

Finally, we examined expression of the genes potentially associated with the drug resistance (Fig. 3, clusters III and IV) in data available from previous studies on cisplatin sensitive and resistant ovarian cancer cells. Consistently with our results, we found that nine of the 18 genes, *ARRDC4, ST3GAL5*, *SYNE1*, *CXCL8*, *KLF4, HERC5*, *FOSL1*, *OASL* and *PBX1* (fc ≥ 1.5, fdr ≤ 0.05), were differentially expressed by cisplatin sensitive A2780 cells when compared to resistant CP70 line (NCBI GEO ID: GSE28648). Another data set on cisplatin sensitive vs resistant A2780 line (NCBI GEO ID: GSE15709) revealed similar pattern for the genes *ARRDC4* (fc ≥ 1.5, fdr ≤ 0.05) and *ST3GAL5*, *SYNE1, IL8/CXCL8* (fc ≥ 1.5, unadjusted p ≤ 0.05). Interestingly, although *KLF4*, *HERC5* (fc ≥ 2.0, fdr ≤ 0.05) and *FOSL1* (fc ≥ 2.0, unadjusted p ≤ 0.05) were differentially expressed, they had an opposite pattern of expression in comparison to our data (Supplementary Table S5). The detected CpG methylation changes had only modest overlap with the results from previous studies. Comparison to data by Yu *et al*.^31^ and Li *et al*.^7^ revealed eight common genes with DNA methylation changes in the near proximity. Of these only *COL18A1, SECTM1* and *ALDH1A3* were transcribed in our cells, however, in our study these genes were not differentially expressed between the sensitive and resistant cells^7, 31^. Overlap with the genes reported by Zeller *et al*.^8^ included only *FLNC*, which had a CpG site with decreased methylation within 700 bp from the TSS. However, again no changes in the gene expression of this gene were observed in our data.

## Discussion

To investigate the molecular mechanisms associated with development of cisplatin resistant in HGSOC, we exploited an *in vitro* model from patient derived primary tumor cells, cultured as spheroids, and exposed repeatedly to cisplatin to induce resistance. Comparison of the cisplatin sensitive parental line to the established resistant line revealed large amount of changes in CpG methylation. The differentially methylated sites were primarily localized in intergenic and intronic regions of the genome. Decreased CpG methylation levels were observed in the cisplatin resistant line in comparison to sensitive line. Our results from the global analysis are in agreement with previous findings^7, 8, 31^. However, we were not able to identify such common DNA methylation changes between the studies, which would explain the drug resistance. This lack of overlap may derive from the heterogeneous mechanism(s) of drug resistance. Alternatively, the differences in the experimental design may have limited the detection of common methylation changes. Previous studies have utilized either array-based methods, detecting less than 30 K CpG sites or capture based assay, which does not have single nucleotide resolution. In this study we used the RRBS method, which enabled genome-wide analysis the CpG rich regions with single nucleotide resolution. In addition, we focused only on consistent CpG methylation changes of over 20%, found in three biological replicates with FDR cut off 0.05, as biological significance of the small changes in DNA methylation is unclear. Further studies, with additional cell lines and comparable or increased genomic coverage and sensitivity are needed to validate our findings.

Similar to the DNA methylomes, the transcriptomes of the sensitive and resistant lines were different. Integrative analysis revealed CpG methylation changes in functional genomic elements correlating with gene expression changes in overlapping or nearby genes. However, the overlap of transcriptome and DNA methylation changes in general was low, as also previously observed^8^. The CpG methylome changes of both the sensitive and resistant lines in response to 0.6 μM cisplatin (IC_50_ for the sensitive line) were modest and transcriptome changes were not detected. A potential explanation for this could be that the cisplatin response may be mediated through post-translational or other cytoplasmic mechanisms, or alternatively the responding cells were lost from the cultures before sampling for downstream analysis. However, this is unlikely as the exposure of the resistant cells to a higher concentration of cisplatin (7 μM), not tolerated by sensitive cells, revealed large-scale transcriptome changes.

Examination of the transcriptome data revealed a specific subset of genes that were differentially expressed by sensitive and resistant cells for which the difference was either partially reversed or further increased by exposure of resistant cells to 7 μM of cisplatin. Some of the changes were also associated with differentially methylated sites in the promoter regions of the affected genes. Most of these genes have been previously associated with ovarian cancer and several with drug resistance or malignancy, supporting potential importance of these genes as key drivers of drug resistance^14–30, 32–34^. In our model the strongest changes associated with drug resistance were induction of *CYP24A1* and *IL6* in the resistant cells and their reciprocal regulation in response to 7 μM cisplatin. The changes in *IL6* gene expression were accompanied with alterations in several other components of IL6 signaling. Increased expression of IL6 has been linked to ovarian cancer and poor outcome in several studies and has been examined as a potential prognostic marker^32–35^. CYP24A1 again regulates processing of vitamin D with potential importance as an anticancer agent^36^. However, further functional studies are needed to elucidate how the IL6 cytokine, vitamin D signaling, and other products encoded by the genes with opposite or similar pattern, such as *KLF4*, affect the cisplatin sensitivity or resistance.

Interestingly, KLF4 was identified as a putative upstream regulator of the genes with both DNA methylation changes and gene expression changes in resistant cells before and after drug treatment. KLF4 is a well-known transcription factor utilized in reprogramming of differentiated cells back to pluripotent stem cell stage^37^, and has a context dependent function in the modulation of cancer properties and mediates adaptive responses and cellular survival in response to therapies^38^. *KLF4* together with a panel of genes (*ST3GAL5, SYNE1, CXCL8/IL8, HERC5, FOSL1, ARRDC4*) was also detected to be differentially expressed by cisplatin sensitive and resistant cells in data from other studies supporting their potential importance in the regulation of drug resistance.

As indicated in Table 1, most of the putative drug resistance driver genes identified in our study have previously been associated with ovarian cancer. However, several new candidates, such as ubiquitin ligases *HERC5* and *ARRDC4*, were also identified. Furthermore, for many of these genes the functional and clinical significance is still poorly understood. ST3GAL5 and FOSL1 regulate for example cell proliferation and differentiation. FOSL1 is also known to regulate both IL6 and IL8/CXCL8 cytokines. SYNE1 is a structural protein linking the plasma membrane to the cytoskeleton. Further studies are needed to define the function of these genes in ovarian cancer. Additional studies are also required to validate our findings in a larger panel of cell lines and clinical tumor samples. Many of the genes found to be differentially regulated in sensitive and resistant lines, such as IL6 and IL8/CXCL8, are implicated in immune cell functions and signaling. Therefore, it will be interesting to further elucidate, how these signaling molecules may affect the tumor microenvironment and anti-tumor responses.

In summary, our results reveal that, in parallel with large-scale transcriptome changes, cisplatin resistance of ovarian cancer cells is associated with loss of hypermethylation in a high number of CpG sites primarily localized in the intergenic regions of the genome. The transcriptome perturbations in response to 0.6 μM cisplatin treatment of both sensitive and resistant cells were minimal suggesting the importance of post-translational mechanisms in mediation of death or survival of the cells. The response of resistant cells to 7 μM concentration of cisplatin, not tolerated by sensitive cells, revealed transcriptomic changes in potential key drivers of drug resistance. The strongest changes were associated with the reciprocal regulation of *CYP24A1* and *IL6*. The expression of several other components of *IL6* signaling were also altered further supporting the previous observations on the importance of this factor in malignant transformation and development of drug resistance in ovarian cancer. In addition, *KLF4* was identified as a putative upstream regulator of drug resistance in ovarian cancer and together with *ST3GAL5, SYNE1, CXCL8, HERC5, FOSL1, ARRDC4* merits further studies.

## Methods

### The aim, design and setting of the study

The aim of this study was to identify DNA methylome and transcriptomic changes associated with cisplatin resistance by using the latest genome-wide methods. For this purpose, a patient derived spheroid tumor cell line was generated, which was repeatedly exposed to cisplatin to induce drug resistance *in vitro*. DNA methylome and transcriptome perturbations were examined in the parental line and in the drug resistant line before cisplatin challenge and in response to concentrations of 0.6 μM (IC_50_ for the sensitive line) or 7 μM (IC_50_ of the resistant line) of cisplatin. The IC_50_ of the resistant cells was not tolerated by the sensitive cell line.

### Cell lines

Primary cell lines M019i and OC002 originated from patient ascites. M019i cells were collected at interval surgery after primary platinum-taxane chemotherapy, while the OC002 cells originated from primary surgery before chemotherapy. Both the patients received the same treatment: After the primary operation, the patients were treated with three cycles of platinum based neoadjuvant chemotherapy, after which an interval operation was performed for debulking. After this operation, the patients received three more cycles of the same chemotherapy. The patients had rapid progression of HGSOC with a progression free survival (PFS) of 2.4 and 10.1 months, respectively. However, M019i responded well with the therapy after relapse (overall survival 34.3 months) while OC002 had a short overall survival of 12.0 months. The patients were diagnosed with HGSOC at the ages of 63 and 65 years, with stage IVB and IIIC, respectively. They were both Caucasian.

To generate a cisplatin resistant cell lines (M019iCis and OC002Cis) the M019i and OC002 cells were grown with increasing cisplatin concentration up to 2.0 μg/ml (6.6 μM) according to the method described by Tsai *et al*.^39^, which lead to a selection of surviving resistant cells. All the cell lines grew as spheroids in serum-free Dulbecco’s Modified Eagle Medium: Nutrient Mixture F-12 (Lonza) culture medium supplemented with B-27® supplement (Life Technologies), 20 ng/ml EGF (Sigma), and 10 ng/ml bFGF (Invitrogen). M019iCis and OC002Cis cells were treated with cisplatin in every third subculture and left to recover for at least three days before being plated for sample preparation. The response of cell lines to cisplatin was followed with regular assays: cells were plated on 96-well plates 2500 cells/well; cisplatin was added on the following day in final concentrations of 0.01, 0.1, 1.0, 10 and 100 μM; cell viability was measured 72 h after the first treatment using the ATP assay (CellTiter-Glo® Luminescent Cell Viability Assay, Promega) in regular experiments with triplicate wells each. The half maximal inhibitory concentration (IC_50_) value for cisplatin increased from 0.6 μM for M019i and 0.8 μM for OC002 to 7.0 μM for M019iCis (11.7-fold) and 5.0 μM for OC002Cis (6.3-fold). All cell lines have been characterized using exome and RNA sequencing and verified to represent HGSOC (unpublished data).

### Nucleic acid isolation

The spheroid samples were homogenized using a Tissuelyzer disrupter (Qiagen) and RNA was extracted simultaneously with DNA and miRNA from all the samples using the Qiagen AllPrep kit according to the manufacturer’s instructions. The samples were quantified with a Nanodrop 2000 spectrophotometer (Thermo Scientific) and the high quality of RNA or DNA was confirmed with an Agilent 2100 Bioanalyzer.

### Next-Generation Sequencing library preparation

Extracted RNA samples from 200 ng of total RNA were processed for mRNA-seq using the TruSeq mRNA kit (Illumina) according to the kit manual. The libraries were prepared for RRBS as previously described^40, 41^. The starting amount of genomic DNA was 200 ng per sample. The libraries were quantified with Qubit (Life Technologies) and the high sample quality was confirmed with Agilent 2100 Bioanalyzer. The RRBS libraries were sequenced with 1 × 50 bp chemistry and the mRNA-seq libraries with 2 × 100 bp chemistry with Illumina HiSeq2500 Next-Generation Sequencer.

### Analysis of RNA sequencing data

A total of 648,014,768 reads were obtained for the 15 samples. The quality of the sequencing data was analyzed with FastQC^42^ and trimming was carried out using Trim Galore!^43^. After trimming the reads were mapped to the UCSC hg19 human reference genome with TopHat^44^ and Bowtie2^45^. The features were then assigned to genes and counted by using the R software featureCounts^46^. A list of genes, corresponding number of reads aligned to that gene, and reads per kilobase per million mapped reads (RPKM) were calculated to generate normalized count values. Genes with differential expression were found with the EdgeR software^47, 48^ and batch correction for biological replicates was performed. The genes with a minimum fold change (FC) cut-off of ± 1.5 in each paired comparison and a false discovery rate (FDR) ≤ 0.05 were considered significant in the global analysis.

### Analysis of RRBS data

From the RRBS analysis 10,031,026–18,271,982 total reads per sample were obtained. The quality of the raw reads was examined with FastQC^42^. The adapter trimming and filtering of the high quality reads was carried out with Trim Galore! Version 0.3.3^43^. After trimming over 9 × 10^6^ reads per sample were left. The conversion efficiencies were examined by using a lambda DNA spike in control and were above 99%. The reads were mapped into the genome with Bismark version 0.14.5^11^. The version of the human genome used in the analysis was hg19. The mapping efficiencies were 58.90–62.70% and 5,985,284–11,102,164 uniquely mapped reads per sample were obtained. The differentially methylated bases were identified with MethylKit version 0.9.5^12^ and R version 3.1.2. Before comparison the raw methylation calls were filtered by discarding all the bases that had coverage above 99.9 th percentile coverage in each sample. In addition, only the methylation calls having coverage ≥ 10x in each of the biological replicate per condition were included in the analysis. In hierarchical clustering or principal component analysis the sensitive and resistant lines were distributed into separate clusters. The CpG sites with consistent minimum methylation difference of 20% in each of the biological replicates with q-value (qval) ≤ 0.05 were considered significant. This relatively stringent filtering criteria was chosen in order to minimize noise caused by technical and biological variation in the experiment with limited number of biological replicates (n = 3). Furthermore, the biological significance of small changes in DNA methylation is not clear. Several other bioinformatics tools, including Integrated Genome Viewer^13^, USCS Genome Browser^49, 50^, GENE-E^14^, GREAT^15^, ChIPSeek^16^ and Ingenuity Pathway Analysis Tool (IPA®, Qiagen), were utilized in the annotation, integration and in depth analysis of the data.

### Quantitative RT-PCR

To validate the differences observed between the sensitive and resistant cells in the transcriptome analysis Taqman qRT-PCR analysis were performed for *AKR1C1*, *CYP4F11*, *CYP24A1*, *IL6*, *KLF4, MIR205HG*, and *SLC6A14* in M019i, M019iCis, OC002 and OC002Cis cells. The primers and probes were designed using Universal ProbeLibrary Assay Design Center (Roche Applied Science). Expression was determined in triplicate samples using TaqMan qRT-PCR with Applied Biosystems 7900HT instrument. Raw qRT-PCR Ct values were normalized against the geometric mean of PPIA and TBP^17^.

### Western Blot Analysis

For Western blotting, total protein aliquots (20 μg) from cell lysates were separated by 10% polyacrylamide gel and blotted onto nitrocellulose membranes. Rabbit polyclonal anti-human CYP24A1 (Atlas Antibodies, HPA022261), mouse monoclonal anti-human GAPDH (Abcam, Ab9482), and anti-rabbit horseradish peroxidase-conjugated secondary antibody (Dako) were used to evaluate protein expression. The signals were visualized by enhanced chemiluminescence (Thermo Scientific) and quantitated using ImageJ (version 1.47 v). CYP24A1 expression was normalized to GAPDH expression.

### IL6 measurement

To measure the concentration of IL6 produced by the cell lines, 10 000 cells per well in 100 μl of growth medium were plated in duplicate on a 96-well plate. The culture media were collected after one hour incubation, centrifuged to remove cell debris and frozen at −20 °C. IL6 concentrations were measured using ProcartaPlex Human IL6 Simplex with Human Basic Kit (Illumina) according to manufacturer’s instructions. Statistical analysis for qRT-PCR, western blot and IL6 measurements were performed using the statistical software Sigma Stat 3.11 (Systat Software Inc., Chicago, IL, USA).

### Ethics approval and consent to participate

Ovarian cancer cells were collected from patients on the basis of informed consent. The use of all patient-derived material has been approved by (i) the Ethics Committee of the Hospital District of Southwest Finland (ETMK): ETMK 53/180/2009 § 238 and (ii) National Supervisory Authority for Welfare and Health (Valvira): DNRO 6550/05.01. 00.06/2010 and STH507A. All the experiments in this study were performed in accordance with the guidelines and regulations by ETMK and Valvira.

### Availability of data and materials

The datasets are available through the Supplementary Tables S1–S5. The raw data for RRBS analysis is available in the Sequence Read Archive (SRA), NCBI, through accession number: (will be available later).

## Electronic supplementary material

Figure S1

Table S1

Table S2

Table S3

Table S4

Table S5
