# Supplementary material for: DNA methylation and Transcriptome Changes Associated with Cisplatin Resistance in Ovarian Cancer
Source: Sci Rep. 2017 May 4;7:1469. doi: 10.1038/s41598-017-01624-4 (PMC5431431; doi:10.1038/s41598-017-01624-4)
Supplement: Supplementary file 1 — Figure S1 [file 41598_2017_1624_MOESM1_ESM.doc]

**DNA methylation and Transcriptome Changes Associated with Cisplatin Resistance in Ovarian Cancer**

Riikka J. Lund^1,*^, Kaisa Huhtinen^2,*^, Jussi Salmi^1^, Juha Rantala^2^, Elizabeth V. Nguyen^1^, Robert Moulder^1^, David R. Goodlett^1,3^, Riitta Lahesmaa^1*^, Olli Carpén^2*^

^1^Turku Centre of Biotechnology, University of Turku, Turku, FI; ^2^Department of Pathology, Medicity Research Unit, University of Turku and Turku University Hospital, Turku, FI, ^3^Department of Pharmaceutical Sciences, University of Maryland, Baltimore, MD, *Equal contribution.

Corresponding Authors: Riikka Lund and Olli Carpén. E-mail addresses: riikka.lund@utu.fi and ocarpen@utu.fi. Postal address: Riikka Lund, Turku Centre for Biotechnology, University of Turku and Åbo Akademi University, Tykistökatu 6A, Biocity 7^th^ floor, FIN-20520 Turku, Finland. Tel: +358 41 517 1616
